# Morphological and molecular identification of ixodid tick species (Acari: Ixodidae) infesting cattle in Uganda

**DOI:** 10.1007/s00436-020-06742-z

**Published:** 2020-06-13

**Authors:** Stephen Balinandi, Lidia Chitimia-Dobler, Giulio Grandi, Teddy Nakayiki, William Kabasa, Johnson Bbira, Julius J. Lutwama, Deon K. Bakkes, Maja Malmberg, Lawrence Mugisha

**Affiliations:** 1 Uganda Virus Research Institute, P.O. Box 49, Entebbe, Uganda; 2 College of Veterinary Medicine, Animal Resources and Biosecurity, Makerere University, P.O. Box 7062, Kampala, Uganda; 3 Bundeswehr Institute of Microbiology, Neuherbergstrasse 11, 80937 Munich, Germany; 4 Section of Virology, Department of Biomedical Sciences and Veterinary Public Health, Swedish University of Agricultural Sciences, Box 7028, 750 07 Uppsala, Sweden; 5 Gertrud Theiler Tick Museum, Epidemiology, Parasites and Vectors, Agricultural Research Council – Onderstepoort Veterinary Research, Pretoria, 0110 South Africa; 6 Evolutionary Genomics Group, Department of Botany and Zoology, Stellenbosch University, Merriman Street, Private Bag X1, Stellenbosch, 7602 South Africa; 7 SLU Global Bioinformatics Centre, Department of Animal Breeding and Genetics, Swedish University of Agricultural Sciences, Box 7023, 750 07 Uppsala, Sweden; 8 Ecohealth Research Group, Conservation & Ecosystem Health Alliance, P.O. Box 34153, Kampala, Uganda

**Keywords:** Uganda, Cattle, Ixodid ticks, Morphology, 16S sequencing, Species distribution

## Abstract

In Uganda, the role of ticks in zoonotic disease transmission is not well described, partly, due to limited available information on tick diversity. This study aimed to identify the tick species that infest cattle. Between September and November 2017, ticks (*n* = 4362) were collected from 5 districts across Uganda (Kasese, Hoima, Gulu, Soroti, and Moroto) and identified morphologically at Uganda Virus Research Institute. Morphological and genetic validation was performed in Germany on representative identified specimens and on all unidentified ticks. Ticks were belonging to 15 species: 8 *Rhipicephalus* species (*Rhipicephalus appendiculatus*, *Rhipicephalus evertsi evertsi*, *Rhipicephalus microplus*, *Rhipicephalus decoloratus*, *Rhipicephalus afranicus*, *Rhipicephalus pulchellus*, *Rhipicephalus simus*, and *Rhipicephalus sanguineus* tropical lineage); 5 *Amblyomma* species (*Amblyomma lepidum*, *Amblyomma variegatum*, *Amblyomma cohaerens*, *Amblyomma gemma*, and *Amblyomma paulopunctatum*); and 2 *Hyalomma* species (*Hyalomma rufipes* and *Hyalomma truncatum*). The most common species were *R. appendiculatus* (51.8%), *A. lepidum* (21.0%), *A. variegatum* (14.3%), *R. evertsi evertsi* (8.2%), and *R. decoloratus* (2.4%)*. R. afranicus* is a new species recently described in South Africa and we report its presence in Uganda for the first time. The sequences of *R. afranicus* were 2.4% divergent from those obtained in Southern Africa. We confirm the presence of the invasive *R. microplus* in two districts (Soroti and Gulu). Species diversity was highest in Moroto district (*p* = 0.004) and geographical predominance by specific ticks was observed (*p* = 0.001). The study expands the knowledge on tick fauna in Uganda and demonstrates that multiple tick species with potential to transmit several tick-borne diseases including zoonotic pathogens are infesting cattle.

## Introduction

Ticks are associated with significant medical and veterinary health problems globally (Brites-Neto et al. 2015). Ticks are obligate hematophagous ectoparasites, which during feeding on their vertebrate hosts, can cause various clinical manifestations including tissue injury, body paralysis, and sometimes anemia during massive infestations (Giraldo-Ríos and Betancur 2018). Since the turn of the nineteenth century when the first description of a tick-transmitted infection was made (Smith and Kilborne 1893), many tick species are now known reservoirs and vectors of a multitude of pathogens that cause significant morbidity and mortality in both humans and animals. Some of the diseases that have since been described such as East Coast fever and Crimean-Congo hemorrhagic fever are challenging public health, veterinary, and socio-economic threats due to their increasing occurrence, pathogenicity, and economic impact (Adams et al. 2016; Kuehn 2019; Wesołowski et al. 2014).

In Uganda, the overall threat of ticks and tick-borne diseases to public health is not well known, partly due to limited knowledge on the natural diversity of ticks across the country. In fact, the most detailed and nationally representative surveys of tick species in Uganda that involved a variety of animal species were done in the 1970s, or earlier (Matthysee and Colbo 1987; Tukei et al. 1970), while the most recent studies have focused mainly on either specific geographical areas or veterinary aspects (Byaruhanga et al. 2015; Magona et al. 2011; Rubaire-Akiiki et al. 2006; Socolovschi et al. 2007). According to Walker et al. (2014), there are approximately 27 species of ticks infesting domestic animals in Uganda that are of socio-economic, veterinary, and human health importance. With the increasing reports of geographical expansion of many tick species (Gasmi et al. 2018; Leger et al. 2013; Nyangiwe et al. 2017; Raghavan et al. 2019; Sonenshine 2018), it is important that regular tick surveys are undertaken for inventory revisions. In this study, we aimed to identify the species of ticks currently infesting cattle across various agroecological zones of Uganda, as well as to provide a baseline investigation to a larger study on ticks and tick-borne diseases in Uganda (Malmberg and Hayer 2019). In order to achieve a more precise taxonomic classification of ticks in our study, we complemented the traditional morphotaxonomic approach with molecular techniques as recently suggested and applied in some studies (Brahma et al. 2014; Ernieenor et al. 2017; Estrada-Peña et al. 2017; Estrada-Peña et al. 2013). Molecular analyses were also done in order to provide sequence information for those tick species in Uganda that were not yet available in GenBank. We used cattle as sentinels because they can be infested with a variety of tick species (Rehman et al. 2017). In Uganda, particularly, intensity of tick infestation on cattle is high and tick-borne diseases are a major problem to cattle keepers (Ocaido et al. 2009). According to the Uganda Bureau of Statistics (2017), cattle is the most socially and economically important type of livestock in the country. Therefore, contact with cattle and/or their products is potentially among the most important routes through which many people come in direct, or indirect, contact with tick-borne zoonoses in Uganda.

## Material and methods

### Study areas

**Fig. 1:**
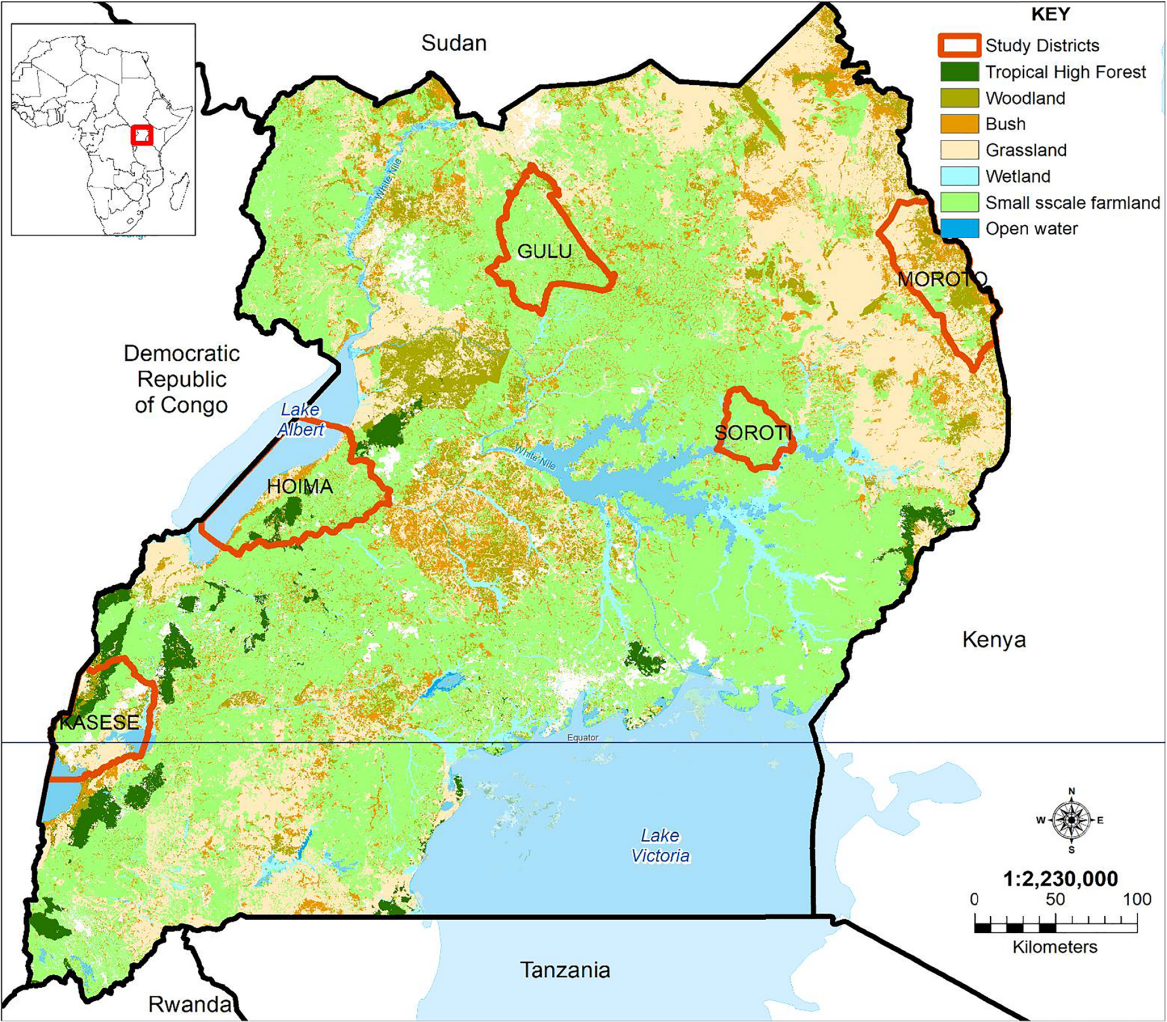
Map of Uganda showing location of study districts (*source*: this map was created using open source data in ArcGIS software, v10.2, Environmental Systems Research Institute, Inc., Redlands, CA, USA)

This study was conducted in the five districts of Kasese, Hoima, Gulu, Soroti, and Moroto in Uganda. As shown in Fig. 1, and based on previous studies by Wortmann and Eledu (1999) and Drichi (2003), these districts represent different agroecological zones of Uganda. Briefly, Kasese and Moroto districts have a semi-arid climatic environment and represent the extreme ends of the Ugandan livestock farming borderlines. Soroti and Gulu lie within a semi-moist zone with scattered subsistence mixed agricultural practices, amidst large swathes of open bushland. These districts are also equidistant to the expansive low-lying swampy areas of Lake Kyoga. On the other hand, Hoima district represented areas with low to medium altitudes that also practice extensive and commercialized agricultural and livestock farming. Additionally, Kasese and Gulu districts border with two major wildlife conservation areas, and therefore are ideal study sites for characterizing ticks at the livestock-wildlife interface. Moroto district represented areas with extensive transboundary migrations of livestock between multiple countries mainly Uganda, Kenya, and South Sudan.

### Study design

This was a cross-sectional study, in which all tick samples were collected between September and November, 2017. To identify animals for sampling, a multistage approach was applied such that in each district, 2 sub-counties were purposively selected based on environmental diversity, and differences in animal management practices. Thereafter, a random selection of one parish from each sub-county was made based on the sampling frame provided by the local administrators. From each parish, 5 villages were identified based on geographical spread. And from each village, 5 households with cattle were selected based on convenience and willingness of the farmer to participate in the study. For tick collection, from each household, two animals were selected from the herd based on the farmer’s choice and/or those with visible ticks on them. Totally, ticks were collected from 100 cattle from each district.

### Tick collection and identification

Ticks were handpicked from one side of the animal’s body, with attention to predilection sites, for approximately 5–10 min per animal. Ticks from each animal were placed separately into 50-ml tubes in which the lid had been perforated with pinholes to allow continuous circulation of fresh air. We also placed 3–4 pieces of fresh grass into each tick-containing tube in an effort to mimic the ticks’ natural environment. All tick-containing tubes were transported in a cool box to Uganda Virus Research Institute (UVRI), Entebbe, Uganda, within 5 days of collection. At UVRI, ticks were identified to species level using morphological characters under a stereomicroscope (Stereo Discovery V12, Zeiss, Birkerød, Denmark) and a Keyence VHX-900 microscope (Itasca, IL, USA) as previously described (Apanaskevich and Horak 2008a; Apanaskevich and Horak 2008b; Voltzit and Keirans 2003; Walker et al. 2014). Representative ticks from each of the identified species and ticks that could not be fully identified at UVRI, were shipped to Bundeswehr Institute of Microbiology, Munich, Germany, to confirm the morphological identification, and where necessary, validate it genetically. For genetic validation, DNA was extracted from individual ticks using a commercially available kit (QIAamp Mini Kit, Qiagen GmbH, Hilden, Germany) according to the manufacturer’s instructions. The 16S rDNA gene was amplified using the polymerase chain reaction protocol as described by Mangold et al. (1998). Thereafter, all obtained sequence data from this study, as well as additional data from GenBank, were compiled into a dataset of 71 sequences. Sequences from GenBank were chosen to encompass the range of *Rhipicephalus* and *Amblyomma* species that occur in Uganda, as well as closely related species. Validity of species identification for these sequences follows from recent studies that include large-scale taxonomic investigations to verify species identity by phylogenetic analysis and correlated morphology (Bakkes et al. 2020; Black and Piesman 1994; Chitimia-Dobler et al. 2017; Dantas-Torres et al. 2013; Nava et al. 2018). The prevalence of misidentified tick species among sequence data in GenBank is a growing problem that can only be addressed by large-scale taxonomic studies. Sequence data were aligned using MAFFT (Q-INS-i, 200PAM/k = 2; Gap opening penalty, 1.53) (Katoh et al. 2002). The optimal nucleotide substitution model was selected using BIC calculations in W-IQ-TREE (Trifinopoulos et al. 2016) and was determined as TPM2+F+G4. Maximum likelihood analysis was performed in MEGA v7.0.14 (Kumar et al. 2016) with 1000 bootstraps, as well as calculation of pairwise p-distances. Average p-distances between conspecific sequences from GenBank and collected samples were calculated to determine species identification validity according to the generally accepted threshold of 5% or greater sequence divergence between species (Bakkes et al. 2020; Bakkes et al. 2018; Chitimia-Dobler et al. 2017; Lado et al. 2018; Li et al. 2018; Mans et al. 2019).

### Statistical analysis

All statistical data analyses were performed in STATA v14.2 software (College Station, TX). Chi-square or Fisher’s exact tests were used as appropriate to compare the differences between tick frequencies obtained from study districts and/or identified species. For all comparisons, a *p* value < 0.05 was statistically significant.

## Results

Five hundred cattle were examined for ticks and only nine (1.8%) were found with no visible tick infestation. Overall, a total of 4362 ticks were collected from cattle in the five studied districts with no significant difference between the total number of ticks collected in each district (*χ*^2^ = 4.0; *p* = 0.40). Altogether, 15 tick species from three genera (*Rhipicephalus*, *Amblyomma*, and *Hyalomma*) were identified. As shown in Table 1, the most dominant tick species collected in this survey were *R. appendiculatus* (*n* = 2259; 51.79%), *A. lepidum* (*n* = 916; 21.00%), *A. variegatum* (*n* = 625; 14.33%), *R. evertsi evertsi* (*n* = 359; 8.23%), and *R. decoloratus* (*n* = 104; 2.38%). Moreover, 4 species including *R. appendiculatus*, *R. evertsi evertsi*, *R. decoloratus*, and *A. variegatum* were found in all study districts, albeit with significant variations in their respective levels of abundance (*p* = 0.001). On the other hand, the least abundant species were *R. simus* and *A. paulopunctatum*—each of them had only a single tick collected from the entire survey.

**Table 1:** Distribution of tick species infesting cattle in Uganda, 2017

Tick species	Study districts					Total	%
	Kasese	Hoima	Gulu	Soroti	Moroto		
*R. appendiculatus* (Neumann, 1901)	604	513	414	545	183	2259	51.79
*R. evertsi evertsi* (Neumann, 1897)	39	1	61	87	171	359	8.23
*R. decoloratus* (Koch,1844)	20	2	18	33	31	104	2.38
*R. microplus* (Canestrini, 1888)	-	-	13	23	-	36	0.83
*R. africanus* (Bakkes, 2020)	-	-	-	-	14	14	0.32
*R. pulchellus* (Gerstäcker, 1837)	-	-	-	-	10	10	0.23
*R. sanguineus* (Latreille, 1806)	-	-	-	-	3	3	0.07
*R. simus* (Koch, 1844)	-	-	-	-	1	1	0.02
*A. lepidum* (Dönitz, 1909)	7	-	-	-	909	916	21.00
*A. variegatum* (Fabricius, 1794)	45	89	182	299	10	625	14.33
*A. gemma* (Dönitz, 1909)	-	-	-	-	8	8	0.18
*A. cohaerens* (Dönitz, 1909)	6	1	-	-	-	7	0.16
*A. paulopunctatum* (Neumann, 1899)	-	-	-	-	1	1	0.02
*H. truncatum* (Koch, 1844)	-	-	-	-	10	10	0.23
*H. rufipes* (Koch, 1844)	-	2	-	-	7	9	0.21
Total	721	608	688	987	1358	4362	100.00
%	16.53	13.94	15.77	22.63	31.13	100.00	

Moroto district had a significantly higher number of tick species (*n* = 13; *p* = 0.004) including all ticks belonging to *R. sanguineus* tropical lineage, *R. pulchellus*, *R. simus*, *A. gemma*, *A. paulopunctatum*, and *H. truncatum*. Importantly, a recently described tick species, *R. afranicus* (formerly *R. turanicus*, see Bakkes et al. (2020)), was also found only in Moroto district. Additionally, 99.23% of all *A. lepidum* in the study was found in Moroto district. On the other hand, Kasese district had 6 species (*R. appendiculatus*, *R. evertsi evertsi*, *R. decoloratus*, *A. variegatum*, *A. lepidum*, and *A. cohaerens*); Hoima district had 6 species (*R. appendiculatus*, *R. evertsi evertsi*, *R. decoloratus*, *A. variegatum*, *A. cohaerens*, and *H. rufipes*), while Gulu and Soroti districts had a uniform distribution of 5 tick species (*R. appendiculatus*, *R. evertsi evertsi*, *R. decoloratus*, *R. microplus*, and *A. variegatum*). Our study highlights identification of *R. microplus* in Gulu and Soroti districts as possible recent expansion and colonization into the area.

**Fig. 2:**
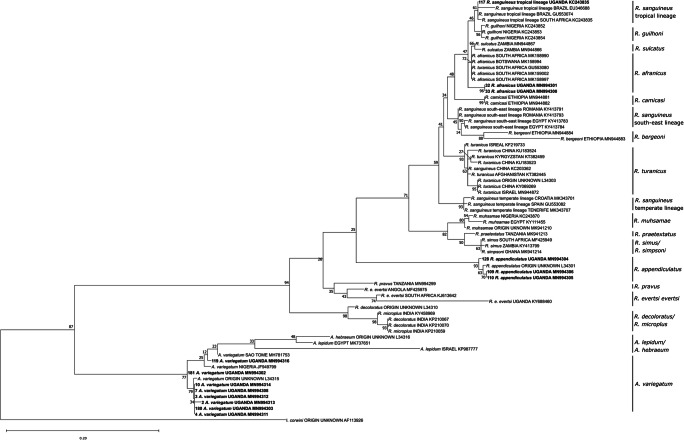
Maximum likelihood phylogenetic analysis of 16S rDNA sequences obtained from ticks infesting cattle in Uganda, 2017, using a TPM2+F+G4 nucleotide substitution model. Indicated are species/lineage and sample names as well as GenBank accession numbers and bootstrap support values. Bold samples refer to sequences generated in this study

*Amblyomma variegatum* specimens (7 females and 6 males) which had been morphologically classified as *Amblyomma pomposum* in Uganda due to their color pattern (especially males), were confirmed genetically as *A. variegatum* with 16S rDNA gene sequencing. Additionally, two *Rhipicephalus* specimens morphologically identified as *R. sanguineus*, were confirmed genetically as *R. afranicus* (male) and *R. sanguineus* (female) tropical lineage. Average pairwise p-distances between conspecific sequences from GenBank versus collected samples were below 5% divergence and supported morphological identification (*R. sanguineus* tropical lineage, 0.7%; *R. afranicus*, 2.4%; *R. appendiculatus*, 0.4%; *A. variegatum*, 2.5%). In summary, from our study, we have generated sequence information for 14 ticks including two sequences belonging to *R. afranicus* that we have deposited in GenBank (Accession numbers: MN994300-MN994317) as shown in Fig. 2.

## Discussion

The main purpose of this study was to identify the species of ticks that infest cattle in Uganda. In total, 15 tick species were identified. *Rhipicephalus* species were the most abundant, among which the commonest species was *R. appendiculatus*. Together with *R. evertsi evertsi* and *R. decoloratus*, they were found across all the study areas. This was followed by *Amblyomma* species, of which only *A. variegatum* was distributed across all study areas. These aforementioned species, together with *A. lepidum* which formed the largest collection from Moroto district, were the major species of ticks found feeding on cattle in Uganda during the study period. The finding of these species in diverse ecological environments has been reported elsewhere (Bazarusanga et al. 2007; Kalume et al. 2013; Sungirai et al. 2015). In fact, the richness of *Rhipicephalus* and *Amblyomma* species in continental Africa is reportedly high (Guglielmone and Nava 2014; Voltzit and Keirans 2003). According to Walker et al. (2014), *R. appendiculatus* covers a more eastern and central African distribution, ranging from South Sudan to the northern parts of South Africa, while *R. evertsi evertsi* is more widespread including parts of West Africa. Similarly, *R. decoloratus* is widely distributed in most areas south of the Sahara, typically within grasslands and wooded areas used as pasture for cattle (Walker et al. 2014). *Rhipicephalus microplus*, an invasive tick species of Asian origin and considered one of the most widespread ectoparasites of livestock, was identified from ticks collected from Soroti and Gulu districts. This is an interesting finding because there have not been any reports of this tick species in Uganda, other than the recent report by Muhanguzi et al. (2020) who morphologically and genetically confirmed its presence in one subcounty of Serere district, south-eastern Uganda. Therefore, taking into account its high dispersal rate as reported in Southern Africa (Nyangiwe et al. 2017), the finding of *R. microplus* in our study, collectively with the findings of Muhanguzi et al. (2020), warrants further investigation about its distribution as a major component of the tick fauna in Uganda. In many countries, so far, the economic costs associated with the control of *R. microplus* is already high (Grisi et al. 2014; Rodriguez Vivas et al. 2017).

The above rhipicephaline distribution in our study was almost mirrored by *Amblyomma* species, with *A. variegatum*, as the most widespread member of this genus as previously reported (Matthysee and Colbo 1987). According to Voltzit and Keirans (2003), and by Walker et al. (2014), in most of the tropical and subtropical Africa, *A. variegatum* has a northern borderline that stretches from Senegal to Ethiopia, and a southern borderline that covers parts of Namibia, through Zambia, northern Zimbabwe, Botswana, and northern Mozambique.

In this study, we noted significant differences in the levels of abundance among the tick species obtained from the different study districts, perhaps depicting the differences in the geoclimatic conditions between the areas. Our study was performed from September to November, which generally in Uganda is rainy, and humid in many parts of the western, central, and eastern regions, and dry in the north-eastern Karamoja region where Moroto district is located (Funk et al. 2012). In particular, *R. appendiculatus* and *A. variegatum* were less abundant in the drier Moroto district, while appearing commonly in the moist and humid district of Soroti in the eastern region. Using a GIS-based model that was supplemented by actual specimen collection, Lynen et al. (2007) observed that *R. appendiculatus* and *A. variegatum* share the same ecological range in Tanzania, being more abundant around the humid lake regions and largely absent in dry areas. This could be associated with their relatively short three-host life cycle that tends to avoid desiccation and long diapause situations (Randolph 1993; Solomon and Kaaya 1998). Conversely, *R. evertsi evertsi* and *A. lepidum* were most abundant in Moroto district, with *A. lepidum* almost exclusively found in this district. Both species are known to have a preference for arid conditions as recently observed in South Africa (Yawa et al. 2018). On the other hand, *R. decoloratus* was almost uniformly distributed across all the study areas reflecting its wide distribution in most of Africa (Walker et al. 2014), with capability to survive at various elevations during wet and dry conditions throughout the year (Abera et al. 2010). However, unlike in the recent findings of Muhanguzi et al. (2020) who concluded that *R. decoloratus* has been displaced by *R. microplus* in Serere district, we found both tick species in sympatry in the neighboring districts of Soroti and Gulu. Although more investigations are necessary to further understand the ecological relationship between these two tick species in Uganda, we think that the displacement process of one species by another in a natural setting is gradual, hence the finding of both species in the same habitat at one point in time. Other tick species, such as *R. simus*, *R. pulchellus*, *A. gemma*, and both *Hyalomma* spp., were less frequent, mainly restricted to Moroto district as similarly described in a previous study (Matthysee and Colbo 1987).

We used molecular tools to correct any morphological misidentifications, as well as to elucidate on the biosystematics of some tick species in Uganda. Herein, we confirm that *A. pomposum*, previously not described in eastern Africa, was not identified in our study, contrary to what was considered from the morphological identification. According to Cumming (1999) and Walker et al. (2014), *A. pomposum* is restricted to parts of Southern-Central African region including Angola, Zambia, and western Democratic Republic of Congo (DRC). We attempted to expound on the biosystematics of *R. sanguineus* in Uganda. As previously reported, the *R. sanguineus* complex includes species with very similar morphology which can easily be misidentified (Chitimia-Dobler et al. 2017; Dantas-Torres et al. 2013; Nava et al. 2012). Consequently, there are wide-ranging nomenclatural and identification ambiguities in this group of ticks (Nava et al. 2015), and a description by their divergent genetic lineages, rather than by the assigned species’ names, has been proposed (Chitimia-Dobler et al. 2017; Nava et al. 2015). So far, at least three lineages, namely, tropical, temperate, and south-eastern lineages, have been identified (Chitimia-Dobler et al. 2017; Nava et al. 2012), but their geographical spread around the world is not well known. Moreover, major differences in the ecology, vector competence, crossbreeding, and other biological attributes of these lineages have also been observed (Eremeeva et al. 2011; Labruna et al. 2017; Levin et al. 2012; Zemtsova et al. 2016). Therefore, a well-documented distribution of *R. sanguineus* lineages is needed. From our study, we confirm that some ticks of the *R. sanguineus* complex in Uganda belong to the tropical lineage. This lineage also includes ticks from South America, Sub-Saharan African, and parts of Southern Asia (Dantas-Torres et al. 2013). Furthermore, we expand on the recently resolved biosystematics of African *R. turanicus* for which a new name, *R. afranicus*, has been proposed (Bakkes et al. 2020). This taxon was recently described as a distinct species that was previously confounded with the name *R. turanicus* in Afrotropical regions (Bakkes et al. 2020). Sequence data for the 16S rDNA gene corroborate separate species status between Africa and the Palearctic (Fig. 2). Within Africa, Ugandan *R. afranicus* showed an average of 2.4% sequence divergence from Southern African samples (Fig. 2), indicating that two distinct populations of this species may exist between Southern and East Africa.

Overall, our findings are similar to what has been observed in recent tick surveys in Uganda (Byaruhanga et al. 2015; Magona et al. 2011; Muhanguzi et al. 2020; Rubaire-Akiiki et al. 2006), as well as in nearby Rwanda (Bazarusanga et al. 2007), Tanzania (Lynen et al. 2007), DRC (Kalume et al. 2013), and Zimbabwe (Sungirai et al. 2015). Interestingly, Byaruhanga et al. (2015) obtained similar frequencies in Uganda for *A. variegatum*, *R. appendiculatus*, *A. gemma*, and *R. pulchellus* as in our study, an indication of their possible endemic stability in the country.

However, our study was limited by the cross-sectional nature of its design as the density of many tick species can vary considerably depending on the prevailing bioclimatic factors (Estrada-Peña et al. 2013). Nevertheless, it demonstrates the high diversity and abundance of multiple tick species infesting cattle in Uganda, thereby raising the potential for the existence of numerous tick-borne zoonoses, perhaps, beyond those that are already known in the country. In fact, in several recent reviews (Brites-Neto et al. 2015; Oguntomole et al. 2018; Shi et al. 2018; Vandegrift and Kapoor 2019), many tick species identified in this study, such as *A. variegatum*, *H. rufipes*, *H. truncatum*, *R. sanguineus*, *R. afranicus*, *R. decoloratus*, and *R. microplus*, are cited as known vectors of a multitude of tick-borne infections in various places around the world, a majority of which are known zoonoses, or suspected to be of zoonotic potential. However, the actual prevalence of these disease agents needs to be determined in order to establish proper public health actions in Uganda.

## Data Availability

Sequence information for the 16S rDNA gene for 14 ticks sequenced in this study have been deposited in GenBank (Accession numbers: MN994300-MN994317). Selected ticks from this study representing the different species have been deposited at Uganda Virus Research Institute Tick Museum, Entebbe, Uganda.
